# Non-Invasive Antibody Assessment in Saliva to Determine SARS-CoV-2 Exposure in Young Children

**DOI:** 10.3389/fimmu.2021.753435

**Published:** 2021-10-08

**Authors:** Constanze Heinzel, Yudi T. Pinilla, Käthe Elsner, Evelyn Friessinger, Benjamin Mordmüller, Peter G. Kremsner, Jana Held, Rolf Fendel, Andrea Kreidenweiss

**Affiliations:** ^1^ Institute of Tropical Medicine, University Hospital Tübingen, Tübingen, Germany; ^2^ German Center for Infection Research (DZIF), Partner Site Tübingen, Tübingen, Germany; ^3^ Centre de Recherches Médicales de Lambaréné, Lambaréné, Gabon; ^4^ Department of Medical Microbiology, Radboud University Medical Center, Nijmegen, Netherlands

**Keywords:** SARS-CoV-2, saliva, antibodies, children, epidemiology, prevalence

## Abstract

Saliva is a body fluid with hitherto unused potential for the assessment of SARS-CoV-2 antibodies. Specific antibodies can indicate a past SARS-CoV-2 infection and allow to estimate the proportion of individuals with a potential protective immunity. First, we carefully characterized plasma samples obtained from adult control groups with and without prior SARS-CoV-2 infection using certified reference ELISAs. Simultaneously collected saliva samples of confirmed convalescent and negative individuals where then used to validate the herein newly developed ELISA for the detection of SARS-CoV-2 IgG antibodies in saliva. The saliva ELISA was applied to assess SARS-CoV-2 exposure in young children (N = 837) in the age between 1 and 10 years in Tübingen, Germany, towards the end of the first pandemic year 2020. Sensitivity and specificity of the new saliva ELISA was 87% and 100%, respectively. With 12% of all Tübingen children sampled *via* their respective educational institutions, estimates of SARS-CoV-2 antibody prevalence was 1.6%. Interestingly, only 0.4% preschool kids were positive compared to 3.0% of primary school children. Less than 20% of positive children self-reported symptoms within two months prior to saliva sampling that could be associated - but not exclusively - with a SARS-CoV-2 infection. The saliva ELISA is a valid and suitable protocol to enable population-based surveys for SARS-CoV-2 antibodies. Using non-invasive sampling and saliva ELISA testing, we found that prevalence of SARS-CoV-2 antibodies was significantly lower in young children than in primary school children.

## Introduction

The COVID-19 pandemic has been keeping the world on its toes for more than one year. Many countries have - and continue - to experience repeated periods of lockdown, and restrictions of their social and economic life. Restrictions and closures of day-care facilities and schools can adversely impact a child’s educational opportunities, supports and overall well-being. From early on, the children´s role in the COVID-19 pandemic was a matter of intensive debate, as there was a lack of relevant data on this novel disease. Fortunately, evidence accumulated relatively rapidly that children and adolescents without underlying chronic disease are rarely suffering from severe clinical manifestations and case fatality is very low (1). However, the contribution of minors to SARS-CoV-2 transmission is difficult to assess and depends on many parameters that vary over time and situational contexts. In 2020, the first pandemic year, children were reported to be less likely to become infected with SARS-CoV-2 compared to adults and elderly. For infants, the reported number of cases were even lower (2–4).

Public records on SARS-CoV-2 cases do not fully inform on the infection rate in children, as PCR testing is largely performed on symptomatic individuals, who are mostly adults. In contrast, children who contract SARS-CoV-2 are commonly mild diseased or even asymptomatic (5–7). Antigen tests have become more widely used since the start of 2021 and might unveil more infections in paediatric populations. Seroprevalence studies detecting antibody responses to SARS-CoV-2 as a marker of a preceding infection, allows for the estimation of the proportion to which the population has been infected (and/or vaccinated). This aids in monitoring of the pandemic´s progression and provides an approximate quantity of individuals with potential protective immunities. Although antibody concentrations are typically assessed in blood, it is known that participants are often reluctant to phlebotomy if not medically required – particularly young children and their parents. An alternative approach is to measure the SARS-CoV-2 specific antibody response in saliva. Sampling of salivary specimens is non-invasive, feasible in non-medical settings, is well tolerated by children and their parents and allows for mass screening. Studies to detect antibodies in saliva have been successfully used to monitor exposure to Norovirus (8), HIV (9) and also malaria parasite infections (10), as these antibody profiles match well with those found in blood. The first SARS-CoV-2 studies conducted confirm this also for previous SARS-CoV-2 infections as well as COVID-19 mRNA vaccination (11–13). Although concentrations in blood are considerably higher (14), salivary antibodies are in fact reactive to the SARS-CoV-2 spike protein (S protein) and to the receptor-binding domain (RBD). Interestingly, both S protein and RBD-specific IgG profiles are more consistent and higher in titres compared to secretory IgA in salivary samples and SARS-CoV-2 IgGs are maintained at least for three months (15). No standardized protocol or certified commercial enzyme-linked immunosorbent assay (ELISA) for the detection of SARS-CoV-2 antibodies in saliva is currently available (by May 2021).

We describe here the development of an in-house ELISA for the detection of SARS-CoV-2 RBD reactive IgG antibodies in saliva, the ‘saliva ELISA’. Assay performance was validated using paired plasma and saliva samples obtained from adult control groups, with their plasma samples carefully characterized using certified reference ELISAs. The saliva ELISA was then applied to non-invasively collected saliva samples obtained in autumn 2020 from young children in the age between 1 and 10 years participating in an ongoing prospective, longitudinal study to determine the incidence of SARS-CoV-2 infections in children and adolescents in the University city of Tübingen, Southwest Germany.

## Materials and Methods

### Study Design and Study Population

To establish and to validate the new saliva ELISA protocol, plasma and saliva was simultaneously collected from control groups that were adults with a prior SARS-CoV-2 infection (convalescent group) or without prior SARS-CoV-2 infection but collected under the pandemic situation in 2020 (negative group). Previous SARS-CoV-2 infection was confirmed by PCR or ELISA documentation. Banked plasma collected from adults in the years before 2020 served as an additional unexposed, negative control (prepandemic group).

Prevalence of SARS-CoV-2 antibodies was assessed in saliva collected in autumn 2020 in frame of the Coro-Buddy study from children aged 1 to 6 years (preschool cohort) and from children 6 to 10 years old (primary school cohort). Additionally, saliva from (mainly) accompanying parents and tutors was collected (adult cohort). Participants or participant´ parents and their household members were surveyed using a questionnaire for positive SARS-CoV-2 tests 6 months prior to sampling and for COVID-19 associated symptoms (fever > 37.5°C, sore throat, cough, rhinitis, others) occurring 2 months prior to sampling. From the few individuals of the adult cohort tested positive for salivary SARS-CoV-2 IgG, blood and an additional saliva sample were collected to reconfirm SARS-CoV-2 IgG positivity by EUROIMMUN ELISA (plasma) and by saliva ELISA. Sample size for the children cohort was based on the assumption of an overall incidence of 509/100.000 in the larger region (Landkreis Tübingen) in April 2020. Randomization was not applicable.

Coro-Buddy is a currently ongoing (by May 2021) observational, prospective study with the overall aim to longitudinally assess prevalence of SARS-CoV-2 antibodies in saliva as a proxy for seroprevalence at three timepoints within 12 months in 1,850 children aged 1 to 18 years in Tübingen, Germany. Here, only the results for the young children (1 to 10 years old) and from adults are reported. Coro-Buddy study protocol includes an additional control group of adults who were previously infected with SARS-CoV-2.

### Sample Collection and Processing

Blood (9 ml) was collected using lithium heparin monovettes and obtained plasma was stored at -20°C. Saliva was collected by Oracol S14 saliva collection device (Malvern Medical Developments, UK) by gently brushing the gum line for 2 min or by drooling into a simple plastic tube (multi-purpose containers 30 ml Greiner Bio-One ref. 201150). Saliva samples were kept on ice for a maximum of 3 hours before being further processed. Oracol S14 saliva collection device and plastic tubes were centrifuged at 2500 rpm for 6 to 10 min, respectively, to remove any debris. Supernatant was transferred into a 2 ml microtube and inactivated solvent/detergent treatment by using tri-n-butyl phosphate (TnBP) and Triton X-100 at final concentrations of 0.3% and 1%, respectively. Samples were stored at -20°C.

### Antigen Expression

The pCAGGS plasmid encoding for the SARS-CoV-2 receptor binding domain (RBD) protein was kindly provided by Florian Krammer (Icahn School of Medicine at Mount Sinai, New York) (16). The RBD sequence encodes for amino acids R319 to F541 of the spike protein plus a C-terminal His tag. Recombinant RBD protein has a molecular weight of 27.5 kDa (without glycosylation). Human embryonic kidney Expi293F™ Cells (HEK cells, Thermo Fisher; ref. A14528) cells were transfected with pCAGGS plasmid using ExpiFectamine™ 293 Transfection Kit (Gibco™; ref. A14525). Transfection and supernatant harvest were performed according to the manufacturer’s manual. Supernatant was purified by an ÄKTA chromatography system using a HisTrap HP 5 ml column (GE Healthcare, ref. 17524802). Protein size and quality control of the recombinant RBD protein were performed by SDS-Page and Western blot analysis. RBD protein concentration was diluted to 2 mg/ml in 1x PBS supplemented with protease inhibitors (Roche; ref. 11697498001).

### ELISA for Saliva Samples

To measure SARS-CoV-2 IgG antibody concentrations in saliva, a new ELISA was established. Prior to the final assay conditions the following blocking reagents were tested: The Blocking solution (Candor; ref. 11001L), Smart Block (Candor; ref. 113125), Plate Block (Candor; ref. 112125), 5% non-fat dried milk in 1x PBS (Roth; ref. T145.2), 1x ROTI Block buffer (0.5x in ddH2O, by Roth; ref. A151.2), Thermo Scientific Blocker Casein in PBS (Thermo Scientific; ref. 37528), Thermo Scientific Pierce Protein-Free (PBS) Blocking Buffer (Thermo Scientific; ref. 37572), gelatin from cold water fish skin (Sigma-Aldrich; ref. G7041-100G), 3% BSA in 1x PBS (SERVA; ref. 11930.04). The final assay conditions were as follows: SARS-CoV-2 RBD antigen was diluted in 1x PBS to a final concentration of 2 μg/ml and 50 μl was added per well to coat Costar 96 well microtiter high binding plates (ref. 3590, Corning). After overnight incubation at 4°C, the wells were washed once with 1x PBS and blocked with 200 µl of The Blocking Solution (Condor Bioscience GmbH) for 2 hours at room temperature on a microplate shaker (700 rpm). Subsequent washing steps were repeated 3x with PBS/0.1% Tween20. Saliva and control samples were diluted 1:3 to 1:27 using The Blocking Solution and 100 μl was added per well and incubated for 1 hour. IgG antibody capture was detected by 1:20,000 diluted biotinylated anti-human IgG (ref. 109-065-008, Jackson Immuno Research Laboratories) and 1:1000 Avidin-HRP (Biolegend ref. 405103). Both reagents were dissolved in 1x ROTI Block buffer (Roth) and incubated for 1 hour and 30 min, respectively. For visualization, 100 µl TMB substrate solution was added, and the reaction was stopped using 50 µl 1M HCl. The plate was read at 450 nm and 620 nm with a microplate reader (CLARIOstar, BMG LABTECH). Concentrations (ng/ml) of salivary SARS-CoV-2 IgG were estimated by comparison against highly pure human IgG (standard) which was precoated as dilution series on separate wells on the same plates (ref. 31154, ThermoFisher). This approach has previously been reported by others (17, 18).

### ELISA for Plasma Samples

Plasma samples were analysed using the EUROIMMUN SARS-CoV-2 IgG ELISAs kit detecting antibodies binding to SARS-CoV-2 Spike protein domain S1 in accordance with the manufacturer’s instructions. Plasma was diluted in a provided sample buffer, added to antigen-coated microtiter wells, and then incubated at 37°C for 1 hour. Plates were washed and then a conjugated solution was added and incubated at 37°C for 30 min. After a second wash step, the substrate solution was added and incubated at RT for 30 min. Finally, 0.5 M sulfuric acid stop solution was added and absorbance of sample wells measured immediately at 450 nm and 630 nm using the CLARIOstar microplate reader (BMG), with output reports generated with optical density (O.D.) at 630 nm subtracted from O.D. at 450 nm. Data were then analysed as recommended by the manufacturer and results reported as a ratio. Following EUROIMMUN specifications the cut-off for SARS-CoV-2 IgG positivity is set at ≥ 1.1 ratio, intermediate antibody concentration is at 0.8 to 1.1 ratio, and negative is defined as < 0.8 ratio. To confirm findings from EUROIMMUN assays, the COVID-SeroIndex, Kantaro SARS-COV-2 IgG Antibody ELISA was performed as second ELISA.

### Data Analysis

Graphics were generated with Graph Pad Prism (Version 9.1) and RStudio (Version 1.2.5001), running R (version 4.0.4.). Proportion of correctly classified samples was calculated for the selection of the cut-off value for the IgG saliva ELISA and ROC-curve analysis was used to describe assay performance and determine the sensitivity and specificity of the test. A Chi²-test was performed to estimate the capacity of the saliva ELISA to discriminate the positive or negative results from the serum ELISAs. The two-way ANOVA trend-test was used to determine if an existing trend for the stability of the saliva specimen at RT, 4°C and multiple freeze-thaw cycles exists. Pairwise Pearson’s correlation coefficient was used to calculate the correlation of saliva samples at RT and 4°C.

Simple descriptive statistics (total number and proportion) were used to describe symptoms, reported previous infections, and prevalence of SARS-CoV-2 antibodies. The estimated prevalence of SARS-CoV-2 antibodies in saliva and its 95% confidence interval were computed and adjusted using the R package epiR Version 0.9-43, which uses the Rogan Gladen estimate for estimating the prevalence and the code provided by Reiczigel et al. (19) for computing the confidence intervals. The adjusted prevalence was calculated considering an imperfect test with parameters of specificity 100% and sensitivity 87%. The prevalence ratio was estimated using the relative risk function implemented in Prism 9.1, and the CI values were estimated using the Koopman asymptotic score.

## Results

### Saliva ELISA Validation

We developed an in-house ELISA to detect IgG antibodies reactive to SARS-CoV-2 RBD domain in saliva, the saliva ELISA. RBD antigen was expressed in HEK cells and a thorough analysis of 9 different blocking reagents was done (**Supplementary Figure 1**). The final protocol is specified in the Methods section.

To characterize the performance of the saliva ELISA to identify SARS-CoV-2 specific antibodies in saliva, a set of adult control groups was selected consisting of paired plasma and saliva samples obtained from i) COVID-19 convalescent individuals (convalescent group) confirmed by either PCR or ELISA documentation (n = 75, median age = 28 years, range of 19-75 years, sex= 51 women/24 men), ii) individuals without known history of SARS-CoV-2 infection (negative group, n = 108, median age = 30 years, range of 13-61 years, sex = 71 women/37 men), and iii) a set of prepandemic plasma samples (but no saliva) collected in the years 2015-2019 (prepandemic group, n = 32, median age = 32 years, range of 23-53 years, sex = 10 women/22 men). None of the COVID-19 convalescent individuals were hospitalized during the disease period. Samples of the convalescent group were collected at 6.5 months (median: 196 days, 95% CI: 185 – 203 days) after symptom onset.

**Figure 1:**
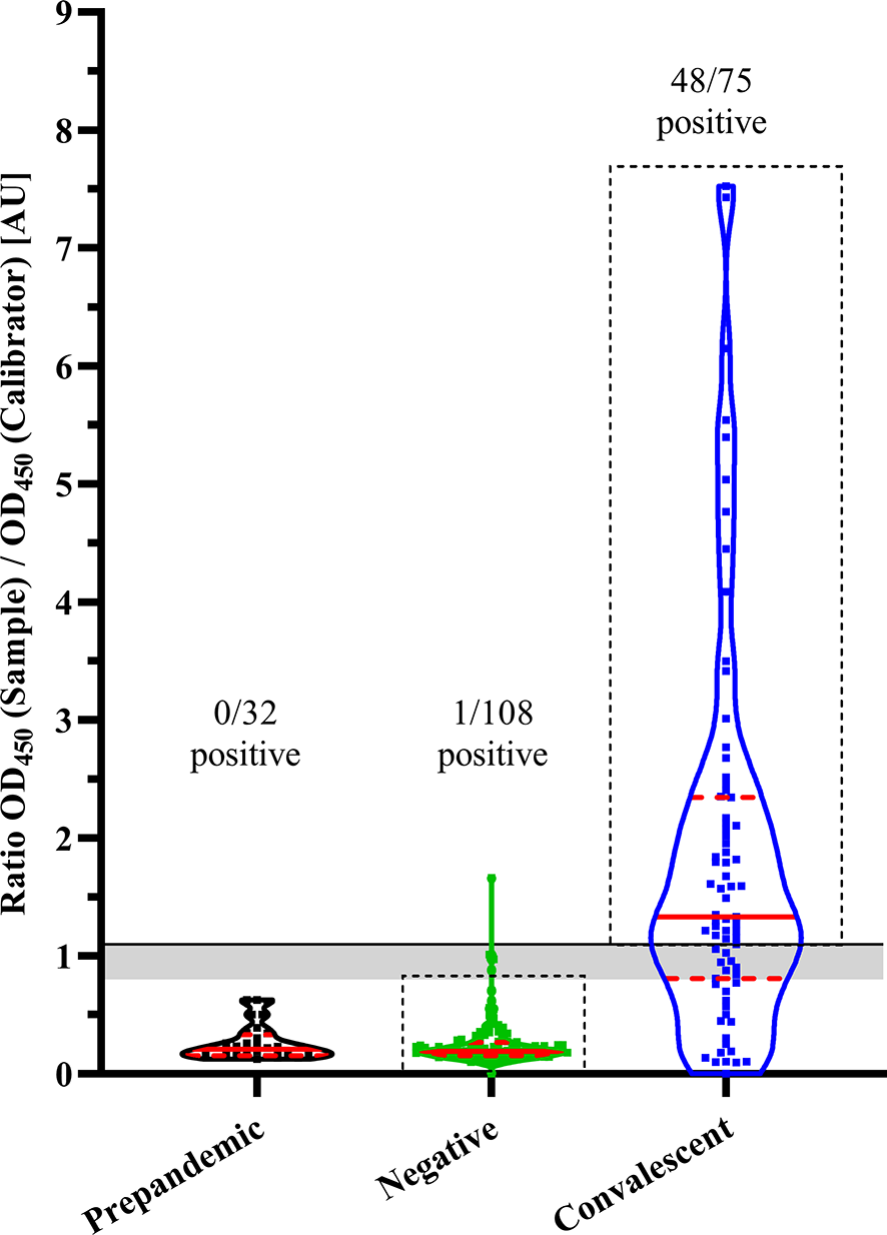
SARS-CoV-2 IgG levels in plasma of adult control groups. IgG concentrations reactive to SARS-CoV-2 were estimated by commercial ELISA (EUROIMMUN). Results are shown as violin plots. Prepandemic: plasma collected 2015-2019, Negative: Individuals without (known) SARS-CoV-2 infection, Convalescent: Individuals with previous COVID-19. Solid line: Cut-off for SARS-CoV-2 IgG positivity; grey area: intermediate antibody concentration; solid red lines: median; dashed red lines: quartiles. Black dashed-line boxes highlight selected individuals for subsequent ELISAs.

Prior to their application for validating the saliva ELISA, plasma samples of the control groups were all confirmed for positivity or negativity by SARS-CoV-2 serology. Therefore, all plasma samples of control groups were assessed by a certified commercial EUROIMMUN ELISA to quantify IgG reactive to the S1-domain of the of SARS-CoV-2 spike protein. Of the convalescent group 48/75 (64%) individuals were plasma IgG positive at the time of sampling (6.5 months after symptom onset). Intriguingly, in the negative group, 1/108 individuals had a positive SARS-CoV-2 IgG titre which was probably due to an asymptomatic, undetected infection (**Figure 1**). No cross-reactivity (0/32) to the S1-domain was found in prepandemic, banked plasma samples. A second ELISA test, COVID SeroIndex detecting RBD domain reactive IgGs, was done to confirm EUROIMMUN results only. Here, 47/48 plasma samples from the convalescent group were IgG positive and 99/104 negative group were confirmed to be seronegative.

Individuals with double-confirmed (EUROIMMUN plus COVID/SeroIndex) positive (convalescent group) or negative (negative group) SARS-CoV-2 serology were selected (**Supplementary Figure 2**), and their saliva samples served to validate the saliva ELISA (see below). Individuals with discordant EUROIMMUN/COVID SeroIndex outcomes were excluded from saliva ELISA performance assessment. For completeness, **Supplementary Tables 1**, **2** display the discordant reference ELISA results and their respective saliva ELISA outcomes.

**Figure 2:**
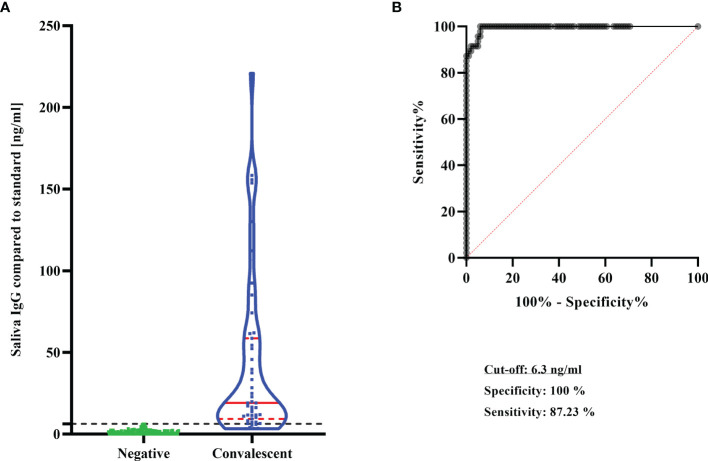
Performance of the saliva ELISA. **(A)** SARS-CoV-2 IgG concentrations in saliva in the negative group (n = 99) and in the convalescent group (n = 47). Results are shown as violin plots. Solid red lines: median; dashed red lines: quartiles, Dashed black line: cut-off of SARS-CoV-2 IgG positivity. **(B)** ROC curve analysis to determine diagnostic test performance of saliva ELISA.

From these double-confirmed control groups simultaneously sampled respective saliva (convalescent group n = 47, negative group n = 99) were used to define the performance of the saliva ELISA. The cut-off for salivary SARS-CoV-2 IgG positivity was determined by the combination of the proportion of correctly classified samples and the highest specificity, which was reached at 6.3 ng/ml. With this cut-off, all plasma confirmed negatives were also negative in the saliva ELISA (99/99). Of the 47 plasma confirmed IgG positives 41 (87%) individuals were found positive for salivary SARS-CoV-2 IgG (**Table 1**). Specificity and sensitivity of the saliva ELISA were 100% (95%CI: 96%-100%) and 87% (95%CI: 75%-94%), respectively (**Figure 2**). The proportion of correctly classified samples was 0.95 (95%CI: 0.91 – 0.98). The area under the ROC-curve was 0.995 (95%CI: 0.988 – 1.0) indicating that the convalescent individuals could be correctly differentiated from the negative individuals with high confidence (p<0.0001). Positive and negative predictive values were above 98% irrespective of the assumed COVID-19 prevalence (**Table 2**).

**Table 1:** Contingency table for saliva ELISA indicating the number of participants used to define the performance of the assay.

Control groups/Saliva ELISA	Convalescent, n (%)	Negative, n (%)
Total, n	47 (100)	99 (100)
Positive, n	41 (87)	0 (0)
Negative, n	6 (13)	99 (100)

Chi²-test (two-tailed): p < 0.0001.

**Table 2:** Saliva ELISA performance characteristics.

Assay parameter			
Sensitivity in % (95%CI)	87.2	(0.75 to 0.94)	
Specificity in % (95%CI)	100	(0.96 to 1.00)	
Predictive values:			
Disease prevalence*:	*0.5%*	*5%*	*10%*
PPV in %	100	100	100
NPV in %	99.9	99.3	98.6
Accuracy in %	99.9	99.4	98.7

*Positive and negative predictive values (PPV, NPV) were calculated for assumed COVID-19 prevalence.

**Figure 3:**
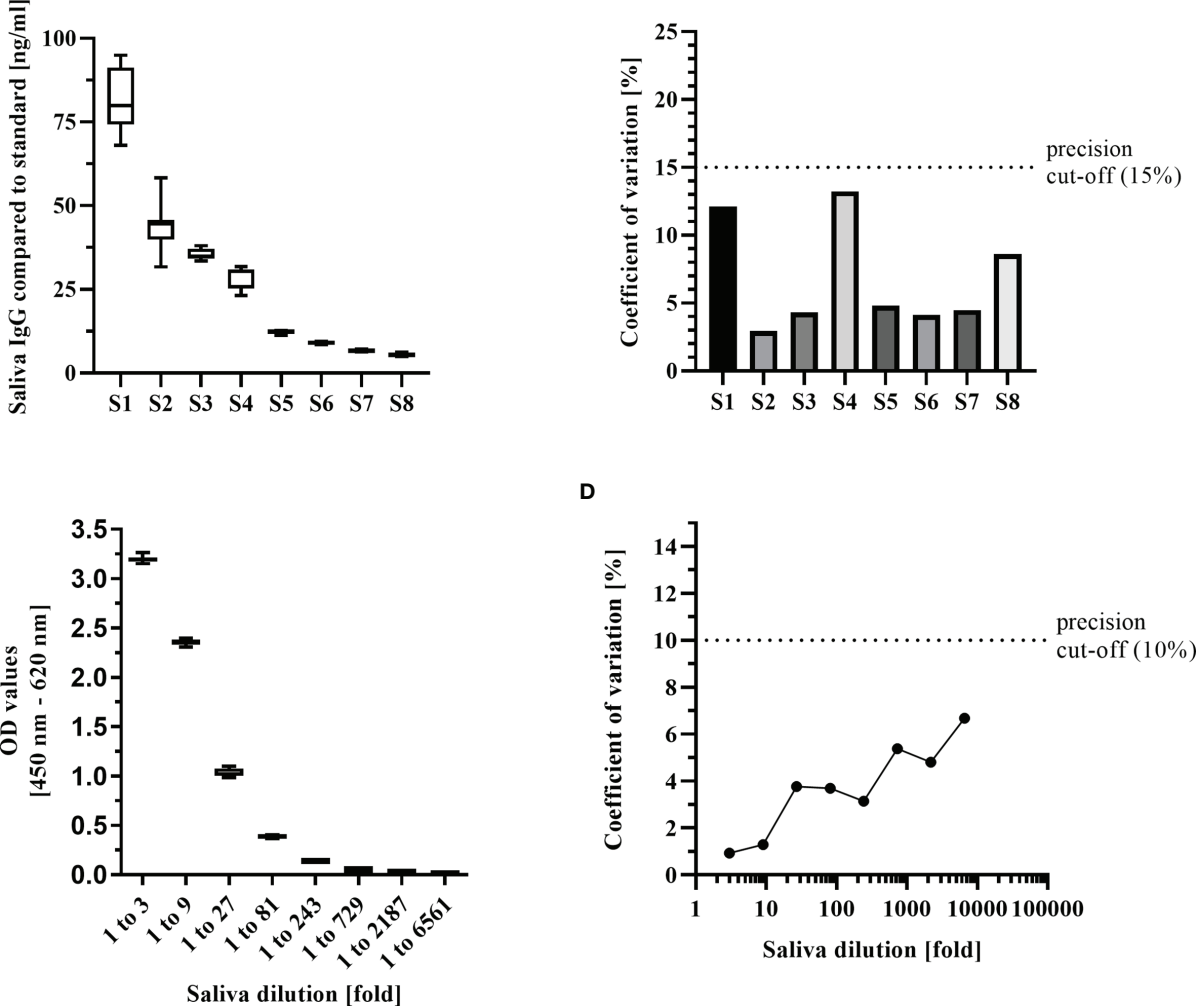
Key performance indicators of the saliva ELISA. **(A)** Inter-assay precision was estimated by measuring 8 samples in triplicates on 3 different days. Samples were selected to represent a range of IgG concentrations from high to negative. Results are shown as boxplots (median with interquartile range, whiskers represent 1.5x interquartile range). **(B)** The coefficient of variation of the inter-assay was calculated for each of the 8 samples. **(C)** Intra-assay precision was estimated by measuring a positive sample in 12 replicates with 8 3-fold dilutions and represented as boxplots. **(D)** Coefficient of variation of the intra-assay was calculated for each of the 8 dilutions.

To determine reproducibility of the saliva ELISA, intra- and inter-assay precision parameters were estimated. For these assays, samples from the convalescent group with salivary IgG covering titres from high (80 ng/ml) to below the positivity threshold of 6.3 ng/ml were chosen. The coefficient of variation (CV) of the inter-assay ranged from 3% -13% which is well below the acceptable 15% for diagnostic assays (20) (**Figure 3**). The CV for the intra-assay repeatability assessed with dilutions of 1 to 3 to 1 to 6561 from a convalescent individual´s sample remained below 10%.

**Figure 4:**
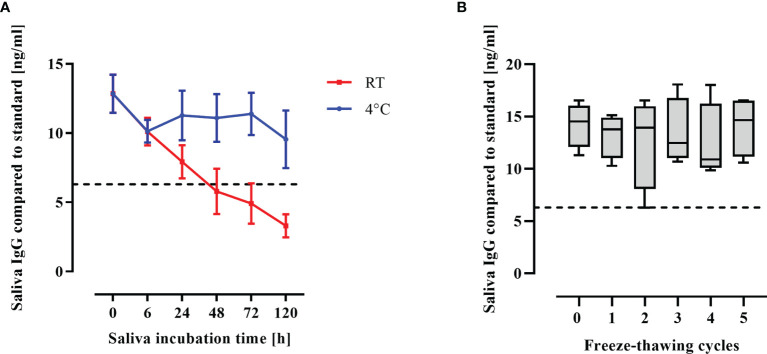
Antibody stability in saliva. **(A)** Saliva was kept at RT or at 4°C for respective durations (h) after collection and SARS-CoV-2 IgG concentrations were measured by saliva ELISA. Mean and standard error of the mean of 4 biological replicates is shown. **(B)** Collected saliva was subjected to freeze-thaw cycles and IgG concentrations were measured by saliva ELISA. IgG concentrations is shown as boxplots. Dashed line: Cutoff for antibody positivity.

Saliva is a complex and non-sterile body fluid. Stability of IgG antibodies during storage and after repeated freeze-thaw cycles was assessed. Saliva samples were stored for up to 5 days either at room temperature (RT) or at 4°C. SARS-CoV-2 IgG remained largely stable when saliva was stored at 4°C (p = 0.1, one-way ANOVA), whereas storage at RT was detrimental to salivary IgG, RBD-specific IgG degraded over time (p<0.0001, one-way ANOVA, test for trend; **Figure 4A**). Therefore, all saliva specimens were stored at 4°C immediately after sampling and frozen after deactivation by solvent/detergent treatment within 6 hours. Freezing and thawing of saliva was not detrimental to salivary SARS-CoV-2 IgG concentrations that remained constant through 5 freeze-thaw cycles (**Figure 4B**).

### Salivary SARS-CoV-2 Antibodies in Children

The Coro-Buddy study is a prospective, longitudinal, observational study on prevalence of salivary SARS-CoV-2 antibodies in children aged 1 to 18 years living in Tübingen, Germany. A total of 1,850 children were enrolled *via* their respective educational institution located in Tübingen, and saliva sampling on site is ongoing *via* the study team. The study began in July 2020 with 3 sampling periods planned - after the first pandemic wave 2020, before winter 2020/2021 and a last sampling period in spring 2021. Here, we report interim data for the prevalence of SARS-CoV-2 antibodies in saliva sampled between September 9^th^ and December 2^nd^, 2020 from 837 children aged between 1 and 10 years old, representing 12% of all children in this age group in Tübingen (**Table 3**, **Supplementary Table 3**). Cumulative SARS-CoV-2 cases (PCR) until early December 2020 in Tübingen were 1.4% (1,249 cases) and 0.5% (34 cases) for the young children only (**Table 4**).

**Table 3:** Characteristics of the study populations.

	Preschool children	Primary school children	Preschool adults*	Primary school adults*
Saliva samples, n	504	333	308	75
Age, median in years (range)	4 (1 – 6)	9 (6-10)	38 (19-62)	46 (20-67)
Previous cold-like symptoms, n (%)	244 (48)	124 (37)	102 (33)	30 (40)
Previous SARS-CoV-2 infection, n (%)	2 (0.4): 2c	2 (0.6): 1b, 1c	5 (1.6): 1b, 4c	1 (1.3): b
Previous SARS-CoV-2 infection in household member, n (%)	13 (2.6): 9b, 4c	8 (2.4): 2a, 1b, 5c	6 (1.9): 1a,1b, 4c	2 (2.6): 1b, 1c
Loss of taste/smelt in household members, n (%)	12 (2.4%)	7 (2.1%)	7 (2.3%)	2 (2.6%)

*Adults, accompanying parents and tutors; a, Reported a positive PCR within 6 months prior to sampling; b, Reported a positive ELISA within 6 months prior to sampling; c, No information provided if prior diagnosis was based on PCR or ELISA.

**Table 4:** Salivary SARS-CoV-2 IgG prevalence and infections within the pandemic year 2020 in Tübingen city.

	Saliva samples, n^§^	Salivary IgG positive, n*	Salivary IgG positivity rate, %*	Estimated salivary IgG prevalence (95% CI)*		PCR positive, n^#^(city)	PCR positive, %^#^(city)
All children	837	12	1.4	1.6	(0.9-2.9)	34	0.5
Preschool children	504	2	0.4	0.5	(0.1-1.7)	15	0.4
Primary school children	333	10	3.0	3.4	(1.9-6.2)	19	0.7
All adults	383	3	0.8	0.9	(0.3-2.6)	1,249	1.4
Preschool adults	308	3	1.0	1.1	(0.4-3.2)	N/A	N/A
Primary school adults	75	0	0	–	–	N/A	N/A

^§^Saliva was sampled between 09 September 2020 and 02 December 2020. *SARS-CoV-2 IgG was detected by the saliva ELISA. ^#^PCR confirmed SARS-CoV-2 cases represent the total cases in the city of Tübingen (not within the study cohorts) and were derived from the weekly reports for Tübingen city reported by the Gesundheitsamt Tübingen that can be obtained via https://www.kreis-tuebingen.de/17094149.html. Data for the children were provided by the Gesundheitsamt Tübingen. The cut-off date was 30 November 2020. Cases in % were either calculated for the total Tübingen population or the respective Tübingen children cohorts. N/A, Not available. Adults were accompanying parents and tutors.

Infants (1 to 6 years) were enrolled in 27 randomly selected preschool facilities and saliva was collected from 504 kids, whereas 333 children (6 to 10 years) were sampled in 6 primary schools. Accompanying parents and tutors were also invited to participate and 308 adults from preschool facilities and 75 from primary schools provided a saliva sample. Roughly 40% in all the cohorts (children and adults) self-reported cold-like symptoms (fever > 37.5°C, sore throat, cough, rhinitis, others) within the 2 months prior to sampling (**Table 3**). A positive SARS-CoV-2 test within 6 months before sampling was self-reported by 2/504 (0.4%) preschool children and by 2/333 (0.6%) of the primary school children. Reported infection rates in adults were slightly higher at 6/383 (1.6%).

**Figure 5:**
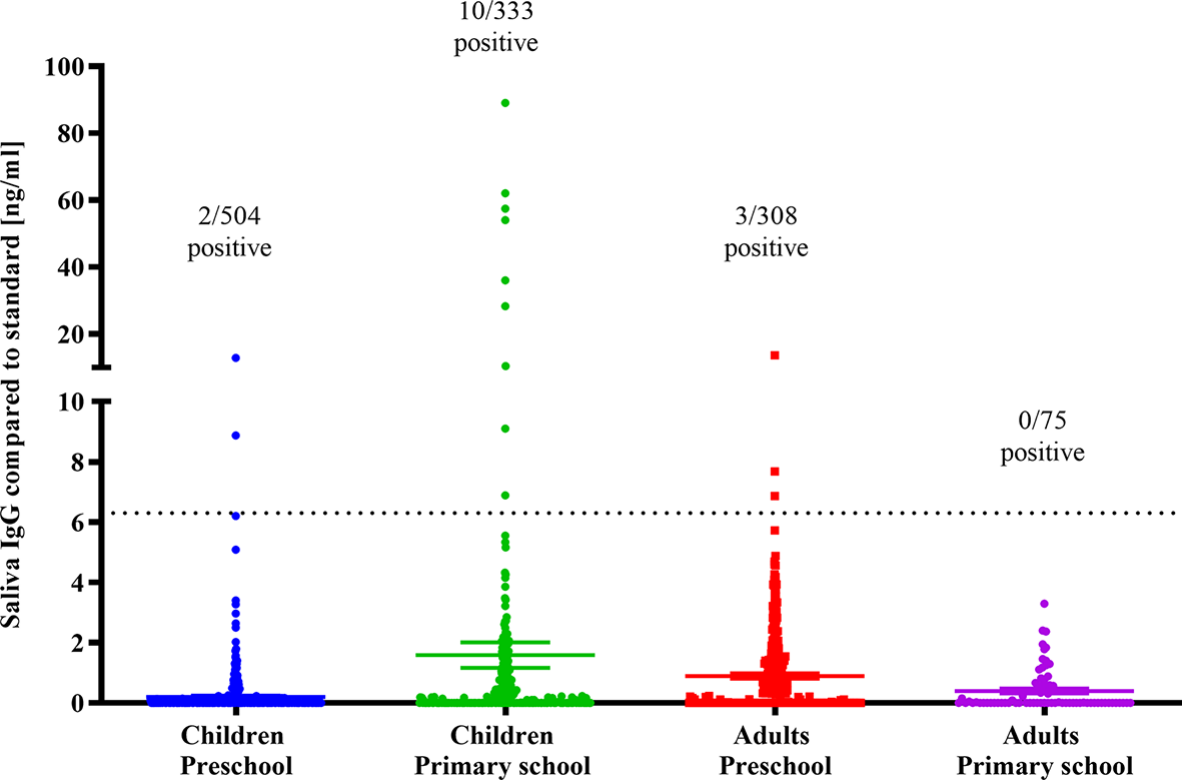
SARS-CoV-2 IgG in saliva of young children and adults. SARS-CoV-2 RBD reactive IgG concentrations in saliva of preschool children and primary school kids and accompanying parents and tutors (adults). Dotted line: Cutoff for SARS-CoV-2 IgG positivity.

Saliva samples were analysed by the established and validated saliva ELISA used to determine positivity rates and titres of IgG specific to the RBD protein of SARS-CoV-2. Estimated prevalence of salivary SARS-CoV-2 IgGs adjusted for the sensitivity and specificity of the saliva ELISA was 1.6% (95%CI: 0.9%-2.9%) for all children, 0.5% (95%CI: 0.1%-1.7%) for preschool kids, and 3.4% (95%CI: 1.9%-6.2%) for primary school children (**Table 4**). Amongst the preschool cohort 2/504 (0.4%) saliva samples were positive for SARS-CoV-2 reactive IgGs with estimated concentrations of 8 to 13 ng/ml (**Figure 5**). Interestingly, 10/333 (3.0%) primary school children had SARS-CoV-2 antibodies, with a median antibody concentration of 12.8 ng/ml (range 6.8 to 89 ng/ml).

The antibody prevalence ratio (PR) of primary school to preschool kids was 7.6 (95% CI: 1.9 – 30.6). Thus, the antibody prevalence was significantly elevated in primary school children compared to the preschool cohort (p = 0.0047, Fisher’s exact test, two-tailed). Out of 12 children with identified salivary IgG during autumn 2020, only 1 child (7 years old) was aware of the infection with one of their household members being infected (**Table 5**). Additionally, 3 children self-reported potential exposure to a SARS-CoV-2 infected household member (2 reported a SARS-CoV-2 infection in a household member and additionally 1 reported loss of taste/smell) (**Table 5**). In addition, of the 12 IgG positives indicating a potential prior infection, only 2 (17%) self-reported cold-like symptoms in the preceding 2 months, whereas the majority (83%) did not report any symptoms.

**Table 5:** Characteristics of salivary SARS-CoV-2 IgG positive individuals.

Participant	IgG titre^#^(ng/ml)	Age (years)	Cohort	Previous SARS-CoV-2 infection*	Symptoms in preceding 2 months*	Previous SARS-CoV-2 infection in household members*	Loss of taste/smell in household members*
1	8.9	2	pre-school	No	No	No	Yes
2	12.9	6	pre-school	No	sore throat	No	No
3	36.1	7	primary	No	No	No	No
4	89.0	9	primary	No	No	No	No
5	28.3	9	primary	No	No	No	No
6	57.5	8	primary	No	No	Yes	Yes
7	62.0	7	primary	Yes	No	Yes	Yes
8	54.0	10	primary	No	No	No	No
9	9.1	9	primary	No	cough, rhinitis	No	No
10	6.9	9	primary	No	No	No	No
11	10.4	8	primary	No	No	No	No
12	10.4	8	primary	No	No	No	No
13	13.4	37	adult	No	No	No	No
14	7.7	40	adult	No	No	Yes	Yes
15	6.9	33	adult	No	No	No	Yes

^#^Saliva IgG compared to standard [ng/ml].

*Self-reported by a structured questionnaire on the day of sampling.

The adult cohort of 383 individuals (0.5% of the adult population in Tübingen) – mostly accompanying parents and tutors - only allowed for limited insights into COVID-19 epidemiology. Estimated prevalence of SARS-CoV-2 antibodies in saliva was 0.9% (95%CI: 0.3%-2.6%) and only 3 (0.8%) persons had SARS-CoV-2 antibodies (6.9, 7.7, and 13.7 ng/ml) (**Table 4**). None of the IgG positive adults were aware of a previous infection, but 1/3 (0.3%) and 2/3 (0.7%) self-reported infection with a loss of taste and/or smell in household members (**Table 5**). From 1 of these 3 salivary IgG positive adults plasma as well as another saliva sample could be sampled 4 weeks later and IgG positivity could be confirmed with EUROIMMUN and with saliva ELISA, respectively. Self-reported SARS-CoV-2 infection was seen for 4/837 children and 6/383 adults (**Table 3**) amongst only 1 individual (child) was also found SARS-CoV-2 IgG positive by saliva ELISA (**Table 5**, participant no. 7).

Of the 9 participants (3 children and 6 adults) who self-reported a previous SARS-CoV-2 infection but were negative for salivary SARS-CoV-2 IgG, 3/9 reported an exposure to infected household members (of note: none of the self-reported SARS-CoV-2 infection was diagnosed by PCR (**Supplementary Table 4**).

## Discussion

In response to the COVID-19 pandemic, we developed a saliva ELISA that facilitates population-based serological surveys for SARS-CoV-2 infections. Asymptomatic patients are often not routinely tested for SARS-CoV-2 infections, in particular the paediatric population, has been underrepresented in epidemiological studies (6). We report here the validation of the saliva ELISA using paired plasma and saliva samples of carefully characterized adults. We then applied the saliva ELISA to estimate exposure of young children to SARS-CoV-2 by early December 2020, during the rise of the second pandemic wave in Germany. The data presented here is part of an ongoing prospective, longitudinal study to follow SARS-CoV-2 antibody prevalence in children aged 1 to 18 years over 3 timepoints within 12 months (summer 2020 to summer 2021). None-invasive saliva sampling is done at respective educational institutions randomly located over the area of Tübingen, a University town in Germany. Estimates for prevalence of SARS-CoV-2 antibodies in young children aged 1 to 10 years was 1.6% and indicated about a 3-fold higher exposure to SARS-CoV-2 compared to the reported PCR diagnosed cases given by the local health department. In accordance with other observations (21, 22), prevalence in preschool children was significantly lower compared to primary school children. Antibody screening revealed the overall extent to which this older cohort had been exposed. Fascinatingly, only 16% of SARS-CoV-2 IgG positive children reported symptoms, while at least 84% of the positives were asymptomatic.

Saliva ELISA testing in the preschool kids and in adults did not identify more cases than known by official PCR records. Therefore, the results for adults should not be overinterpreted as only a small sample of 0.5% of the total adult population in the city was investigated. SARS-CoV-2 antibodies indicate that a person has been infected (and/or COVID-19 vaccinated) and IgGs can be detected in the blood as soon as two weeks after infection (23) and beyond. This is consistent with previous findings (24), where about two-thirds of the mild COVID-19 adult controls still had RBD binding IgG titres 6 months after symptom onset in blood - but also in saliva although at a lower magnitude. SARS-CoV-2 RBD antibodies possess virus neutralization activity but relevant titres and their role in protection from infections is still under investigations. It was interesting to see that COVID-19 vaccination induced SARS-CoV-2 IgG titres in saliva (and to a lesser extent also in IgA) and levels were similar to those found in blood (12, 13). Secreted IgA is produced by plasma cells of the mucosae and associated glands in the oral cavity, whereas IgG in saliva originates mainly from plasma cells residing in the bone marrow, circulating in the blood, before entering the saliva *via* transudation (25). As shown by others and confirmed by our work, SARS-CoV-2 IgG in saliva correlates well with the response in blood, therefore saliva IgG levels may therefore reflect the systemic antibody response. Saliva collection is a convenient alternative to blood sampling that allows for self-sampling and is easy to use in the paediatric population. This is especially important for studies that need to be conducted in non-medical settings without skilled personnel. Self-sampling devices include plastic containers for simple drooling or commercial devices that are built on saliva-absorbent tissues (e.g. Oracol, OraSure, etc.). The latter is particularly useful for the sampling of infants who often have difficulties with controlled spitting. However, the device should be carefully chosen and documented as the collection methodology might impact the antibodies observed. In addition, quantification of specific antibody using the saliva ELISA could be altered by the hydration status or the gum health of the individual, but sensitivity or specificity should not be affected by this.

As of May 2021, there is no ELISA commercially available that is approved for detection of salivary IgG reactive to SARS-CoV-2 RBD; one kit detects IgG binding to the nucleocapsid protein in oral fluids (ref 1-1260, Salimetrics, LLC) that is highly homologous to endemic coronaviruses. The published sensitivity and specificity of the test is similar to the saliva ELISA reported here (92% and 98%, respectively), but the slightly reduced specificity has a strong impact on the positive predictive value in diseases with low prevalence of salivary SARS-CoV-2 antibodies (13). Published studies on salivary SARS-CoV-2 IgG either used advanced immunoassay techniques [Luminex (11), immunoprecipitation systems (26)], in-house ELISAs, or repurposed commercial ELISAs for salivary IgG measurement (27, 28). All work reported a consistent positive correlation between blood and saliva antibodies particularly for RBD specific IgG. Nonetheless, due to the known lower concentration of antibodies in saliva compared to blood (29), all assays required protocol optimizations to increase sensitivity while controlling for an optimal signal-to-noise ratio. The saliva ELISA protocol reported here includes an improved blocking strategy and a simple biotin-avidin signal amplification step. The blocking condition for each of the ELISA steps were optimized, the final protocol uses a different antibody for the blocking of the antigen and the blocking of the secondary antibody. This is unusual but can be explained by the different nature of interaction between the antigen and the saliva/serum antibodies or the interaction of the human antibody and the secondary antibody. To create a saliva ELISA protocol for population-based surveys (and less for patient care), we tailored the test performance towards 100% specificity and accepted a lower sensitivity of approximately 87%. A population-based antibody survey, in the current low prevalent COVID-19 epidemiology, shows that seroprevalence estimates are much more affected by a low test specificity then by a low test sensitivity [e.g. if the ‘true’ COVID-19 prevalence is 5%, the estimated seroprevalence could double when the specificity is ‘only’ 95%, however estimates would decrease by max. 1% when the sensitivity is ‘only’ 80% (30)].

The sensitivity and specificity of the saliva ELISA was determined with stringently selected samples based on two different serum ELISA having the emergency use authorization (EUA) form the FDA. Discordant results from the two assays were excluded from the saliva ELISA validation, as it is not very clear how these samples should be classified. Regarding caveats and limitations, we are still unsure for how long SARS-CoV-2 IgG remain detectable in the saliva of children. For antibodies in the plasma and saliva of adults, titres can be detected for at least 6 months after infection as shown by our results and reports from the literature. Titres in saliva have been reported to be considerably lower compared to plasma and thus, any tests reach its detection limits. Despite little sequence homology of SARS-CoV-2 RBD to endemic coronaviruses (31), cross-reactivity of antibodies induced by seasonal human coronaviruses infections to the RBD saliva ELISA cannot be fully excluded although generally considered minimal (32–34). A study has shown that approximately 1% of prepandemic blood samples from children and adults cross-reacted with SARS-CoV-2 RBD (35), even though the comparison of different studies can be difficult as assay conditions play a pivotal role. In this line, specificity of the saliva ELISA using saliva from children sampled in prepandemic times would be of great value but were not available. Additionally, performance outcomes of the saliva ELISA always depend on the reference serology applied - here on EUROIMMUN sample classification and a different sensitivity or specificity cannot be excluded.

Saliva sampling was done in educational institutions but unless there was an obvious clustering of cases unfortunately, we are unable to provide information regarding the efficacy of school measures to combat the pandemics. It is also difficult to make informed statements in relation to an institution´s methods to limit the spread of SARS-CoV-2, as the influence of constant measures such as school closures and other lockdowns like curfews have significantly reduced transmission rates. It will be of high interest to investigate the prevalence of SARS-CoV.2 antibodies in children and all vulnerable populations in other areas of Europe and around the world.

The saliva ELISA is a valid and suitable protocol to enable population-based surveys for SARS-CoV-2 antibodies, that are needed to monitor SARS-CoV-2 explorations in all age groups. Using non-invasive sampling and saliva ELISA testing, we were able to elucidate that the antibody prevalence in infants and children attending preschool facilities was significantly lower than children attending primary schools.

## Data Availability Statement

The original contributions presented in the study are included in the article/**Supplementary Material**. Further inquiries can be directed to the corresponding authors.

## Ethics Statement

The study protocol was approved by the ethics committee of the University Hospital Tübingen (B312/2020BO1) and registered at Clinicaltrials.gov (NCT04581889). Written informed consent was obtained from adults or the child’s parents or legal guardians if under the age of 18 years.

## Author Contributions

AK, RF, JH, BM, and PK conceived and designed the study. RF, KE, and CH designed and conducted all experiments. YP and EF did the sample collection and sample processing. YP supervised the study conduct. CH, YP, JH, RF, and AK verified and analysed the data. JH, RF, and AK did the statistical analysis. AK, CH, YP,and RF wrote the first manuscript draft. AK, RF, and JH revised and finalized the manuscript. All authors reviewed and approved the contents of the manuscript. All authors contributed to the article and approved the submitted version.

## Funding

The project was supported by the University Hospital Tübingen, Germany.

## Conflict of Interest

The authors declare that the research was conducted in the absence of any commercial or financial relationships that could be construed as a potential conflict of interest.

## Publisher’s Note

All claims expressed in this article are solely those of the authors and do not necessarily represent those of their affiliated organizations, or those of the publisher, the editors and the reviewers. Any product that may be evaluated in this article, or claim that may be made by its manufacturer, is not guaranteed or endorsed by the publisher.
