# Supplementary material for: Non-Invasive Antibody Assessment in Saliva to Determine SARS-CoV-2 Exposure in Young Children
Source: Front Immunol. 2021 Oct 8;12:753435. doi: 10.3389/fimmu.2021.753435 (PMC8531807; doi:10.3389/fimmu.2021.753435)
Supplement: Supplementary file 1 [file DataSheet_1.docx]

**Supplementary files**

**
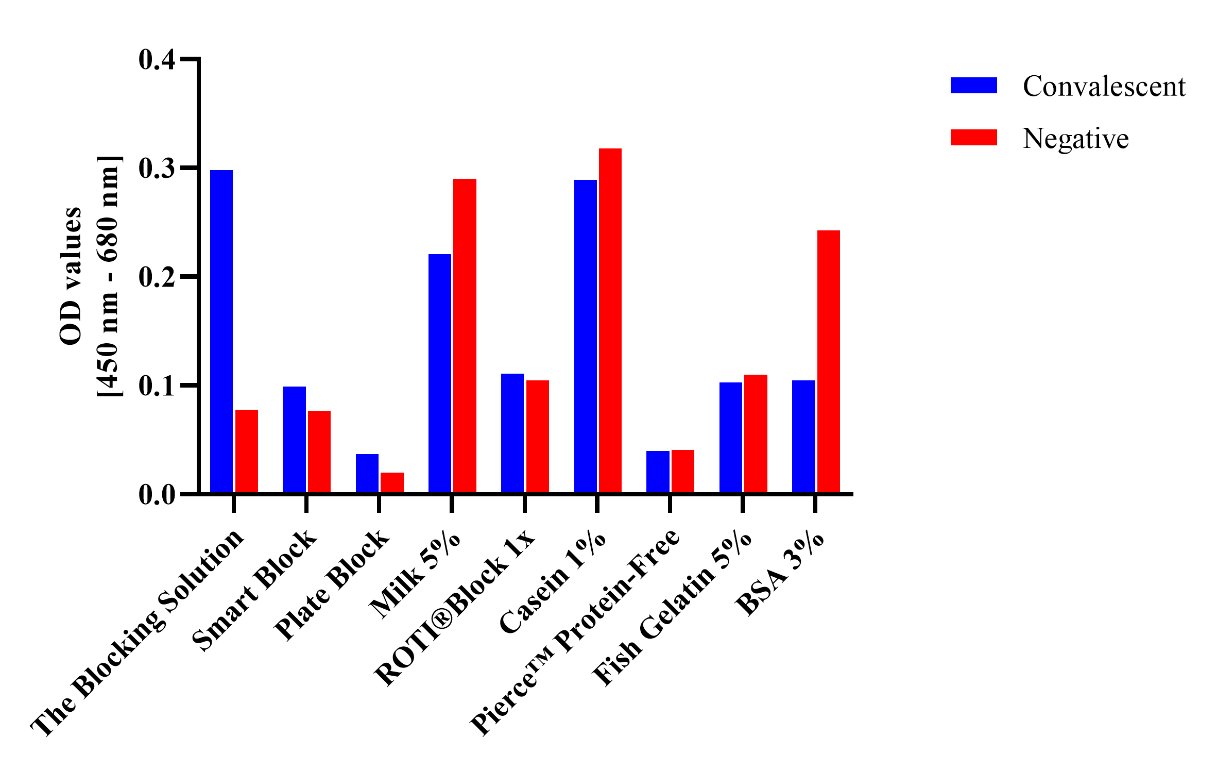
**

**Supplementary Figure 1. Comparing blocking reagents for saliva ELISA.** Commercial and in-house blocking buffers were tested for specific blocking during the development of the RBD-specific saliva ELISA by comparing the OD values between a positive and a negative control. The same buffers were used for both the blocking of the plates as well as the saliva sample dilution. The saliva specimen from the controls were diluted 1 to 2 in the following blocking buffers: The Blocking Solution (Candor); Smart Block (Candor); Plate Block (Candor); 5% non-fat dried milk (Roth) in 1x PBS; 1x ROTI-Block buffer (Roth) in ddH_2_O; 1% Casein Blocker in PBS (Thermo Scientific); Pierce Protein-Free blocking buffer in PBS (Thermo Scientific); 5% Gelatin from cold water fish skin (Sigma Aldrich) in 1x PBS and 3% BSA (SERVA) in 1x PBS.


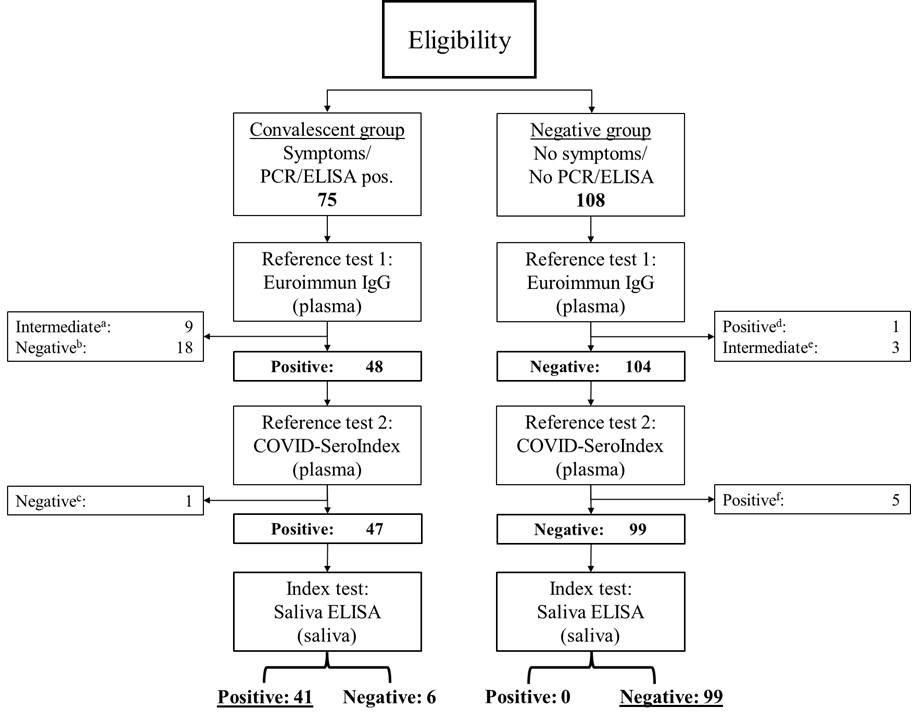


**Supplementary Figure 2. Sample selection flow for saliva ELISA validation.** Plasma samples from the convalescent and the negative control groups were successively tested by two commercial ELISAs for SARS-CoV-2 specific IgG. Of positive and negative individuals, respectively, simultaneously collected saliva samples were used to validate the index test, the saliva ELISA. ^a, b, c^ are classifiers for Supplementary Table 2 and ^d,e,f,^ are classifiers for Supplementary Table 2.

**Supplementary TABLE 1.** Convalescent individuals with discordant reference ELISA results and saliva ELISA outcomes.

| **EUROIMMUN plasma** | **Intermediate^a^** | | **Negative^b^** | | **Negative^b^** | | **Positive^c^** | |
| --- | --- | --- | --- | --- | --- | --- | --- | --- |
|  | **N = 9** | | **N = 9** | | **N = 9** | | **N = 1** | |
| **SeroIndex plasma** | Positive: 9 | | Positive: 9 | | Negative: 9 | | **Negative: 1** | |
| **Saliva ELISA** | Pos: | Neg: | Pos: | Neg: | Pos: | Neg: | Pos: | Neg: |
|  | 5 | 4 | 4 | 5 | 0 | 9 | 0 | 1 |

^a, b, c^ refers to the respective samples displayed in Supplementary Figure 2.

Pos: positive, Neg: negative

**Supplementary TABLE 2.** Negative individuals with discordant reference ELISA results and saliva ELISA outcomes.

^d, e, f^ refers to the respective samples displayed in Supplementary Figure 2.

Pos: positive, Neg: negative

| **EUROIMMUN plasma** | **Positive^d^** | | **Intermediate^e^** | | **Intermediate^e^** | | **Negative^f^** | |
| --- | --- | --- | --- | --- | --- | --- | --- | --- |
|  | **N = 1** | | **N =1** | | **N = 2** | | **N = 4** | |
| **SeroIndex plasma** | Negative: 1 | | Positive: 1 | | Negative: 2 | | **Positive: 4** | |
| Saliva ELISA | Pos: | Neg: | Pos: | Neg: | Pos: | Neg: | Pos: | Neg: |
|  | 0 | 1 | 0 | 1 | 0 | 2 | 0 | 4 |

**Supplementary TABLE 3.** Census of Tübingen city and Coro-Buddy participants.

| **Parameter** | **Tübingen, n** | **Coro-Buddy, n** | **Coro-Buddy, %** |
| --- | --- | --- | --- |
| Total population, n | 91,656 |  |  |
| > 18 years | 78,380 | 383 | 0.5 |
| 0 to 18 years | 13,276 | n/a | n/a |
| 6 to 10 years, n | 2,280 | 333 | 11.8 |
| 1 to 6 years, n | 4,114 | 504 | 12.3 |

Census represents Tübingen administrative city area (city and suburbs) as of 31 December 2019. Data were retrieved and reclassified from <https://www.tuebingen.de/1370.html#/1376/1379>. n/a: Not applicable.

**Supplementary TABLE 4.** Characteristics of salivary SARS-CoV-2 IgG negative individuals who self-reported a previous infection.

| Participant | Age (years) | Self-reported SARS-CoV-2 infection* and how diagnosis was done | SARS-CoV-2 diagnosis  - months before saliva sampling | Symptoms in preceding 2 months* | Previous SARS-CoV-2 infection in household member, n * |
| --- | --- | --- | --- | --- | --- |
| A | 5 | § | 6 | Fever | 3 (A), 1 (Ch) |
| B | 4 | § | 6 | Fever | 3 (A), 1 (Ch) |
| C | 8 | ELISA | 1 | Cough | No |
| D | 44 | § | 6 | Cough, rhinitis | 3 (A) |
| E | 50 | § | 1 | Fever, sore throat, cough, loss of taste | No |
| F | 23 | § | 5 | No | No |
| G | 47 | § | 2 | Fever, sore throat, cough, rhinitis | No |
| H | 43 | ELISA | 5 | Cough, rhinitis | No |
| I | 47 | § | 1 | Rhinitis | No |

*Self-reported by a structured questionnaire on the day of sampling. § No information provided if prior diagnosis was based on PCR or ELISA. A: Adult; Ch: Child
